# Interfacial Intelligence for Polymer Brushes: Toward Physics-Grounded Design

**DOI:** 10.1021/jacsau.6c01349

**Published:** 2026-09-28

**Authors:** Jie Zhu, Xinran Zhao, Dong Wu, Sarah L. Perry, Hao Li, Xiayun Huang, Zhihong Nie

**Affiliations:** † The State Key Laboratory of Molecular Engineering of Polymers and Department of Macromolecular Science, 12478 Fudan University, Shanghai 200438, People’s Republic of China; ‡ Advanced Institute for Materials Research (WPI-AIMR), Tohoku University, Sendai 980-8577, Japan; § Department of Chemical and Biomolecular Engineering, 14707 University of Massachusetts Amherst, Amherst, Massachusetts 01003, United States

**Keywords:** polymer brush, interfacial intelligence, polymer physics, machine learning, antifouling

## Abstract

Polymer brushes are adaptive soft interfaces whose functions emerge from the coupling of molecular chemistry, chain architecture, grafting statistics, solvent and ion organization, and operating conditions. This complexity enables diverse functions in antifouling, lubrication, separations, sensing, and colloidal assembly but also limits the transferability of empirical design rules and purely data-driven predictions. Here, we define interfacial intelligence as a physics-grounded, state-resolved framework in which the realized interfacial state under defined operating conditions connects molecular design to function and is iteratively tested and updated through experiments. We discuss how molecular identity and brush-specific structural variables can be connected with state-resolved measurements, polymer theory, and molecular simulations to represent hydration and ion organization. Using protein-resistant brushes as a recurring example, we examine how regression identifies conditional physicochemical relationships, how high-dimensional analysis identifies candidate interfacial states and interaction patterns, and how molecular representation and literature-based tools connect chemical space to distributed experimental evidence. We further consider how active learning and automated experimentation could use these relationships to guide the next synthesis or measurement. We distinguish predictive association from mechanistic evidence and argue that physics-grounded models should generate testable predictions of how an interfacial state and its function respond to controlled perturbations. Finally, we outline a closed-loop strategy linking provenance-aware data sets, physical models, machine learning, controlled synthesis, state-resolved characterization, and adaptive experimentation. Such integration could shift polymer-brush informatics from end point prediction toward experimentally accountable and transferable interfacial design.

## Graphical abstract

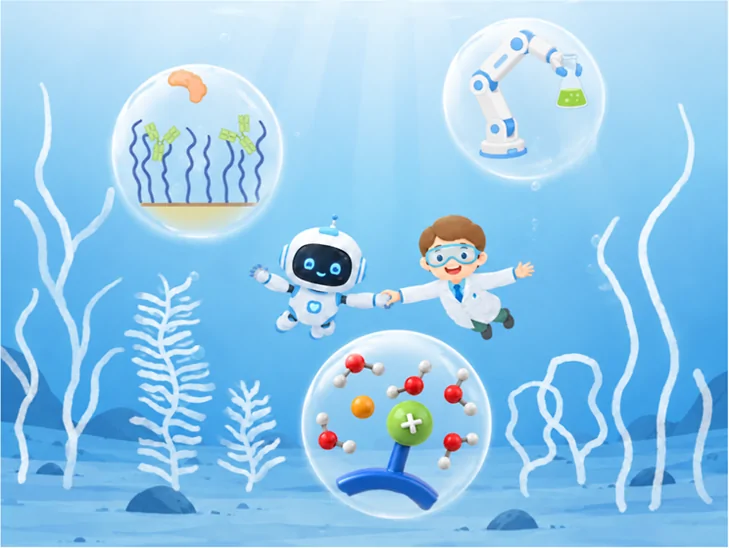

## 1 Introduction

Polymer brushes are surface-tethered polymer architectures in which macromolecular chains are densely attached to a substrate.^1–4^ When neighboring chains overlap, excluded-volume interactions and chain elasticity drive them away from the surface, creating a soft interfacial layer whose structure depends on chain length, grafting density, solvent quality, and, for charged brushes, ion partitioning and charge regulation.^5–8^ This collective behavior enables polymer brushes to regulate adhesion, wetting, friction, molecular partitioning, and transport, supporting applications in antifouling coatings, lubrication, sensing, membrane separations, nanoparticle assembly, catalysis, pyroelectric and piezoelectricity devices, and capacitors.^9–19^ Their function therefore arises not simply from repeat-unit chemistry but from the spatially organized hydration, electrostatic, steric, mechanical, and transport environments generated by tethered chains.^20–26^

The interface encountered during operation is considerably more complex than the descriptors commonly used to characterize a polymer brush. Repeat-unit chemistry, average molecular weight, grafting density, and dry thickness are indispensable descriptors, but they do not uniquely define the environment experienced by a protein, ion, solvent molecule, or opposing surface.^23^ This environment emerges from multiple coupled determinants. Polymer chemistry governs solvation, ionization, hydrogen bonding, and specific interactions, while chain architecture, molecular-weight distribution, grafting statistics, and defects shape brush conformation, segment–density profiles, and relaxation dynamics.^27–30^ Interfacial geometry further modifies these properties through curvature, confinement, lateral heterogeneity, and substrate exposure. Processing history and operating conditions determine how the brush swells, charges, reorganizes, ages, and responds to repeated exposure.^31^ A separate but related challenge arises from the experimental realization of the brush itself. Surface-initiated polymerization, one of the most widely used strategies for synthesizing polymer brushes with controlled structural characteristics, grows polymer chains from surface-bound initiating sites and enables systematic variation of brush thickness and grafting characteristics.^32–34^ However, average quantities alone do not fully describe the resulting brush structure. Incomplete initiation, termination, and spatial variations in chain growth can generate distributions in chain length and grafting density, while the molecular-weight characteristics of surface-grown chains may differ from those of polymers formed simultaneously in solution. Molecular-weight distribution, dispersity, and grafting heterogeneity should therefore be considered integral components of brush structure rather than secondary measures of synthetic quality. These quantities are also experimentally challenging to determine directly for surface-tethered chains, making it important to distinguish measured brush properties from values inferred from solution polymers and idealized structural models.^14,35–37^ Synthetic heterogeneity and environment-dependent reorganization mean that nominally similar brushes may develop distinct wet-state structures, whereas chemically different brushes may converge toward similar functions when they generate comparable hydration, steric, or electrostatic environments. The relevant design target is thus not a nominal composition or any isolated structural descriptor but the physicochemical state realized by the brush under operating conditions.

Protein-resistant interfaces provide a particularly instructive example of why the interfacial state matters.^38–40^ Early design principles emerged from complementary chemical and physical perspectives.^41,42^ Systematic studies of self-assembled monolayers by Whitesides and co-workers associated low protein adsorption with neutral, hydrophilic groups possessing characteristic hydrogen bonding properties, establishing influential molecular heuristics for nonfouling surfaces.^43,44^ In parallel, theories of poly­(ethylene glycol) (PEG)-coated surfaces showed that protein adsorption can be suppressed by the osmotic and elastic penalties associated with compressing or penetrating a tethered polymer layer.^45–48^ These principles motivated the development of dense oligo­(ethylene glycol), PEG, and zwitterionic brushes exhibiting very low fouling in protein solutions, serum, and plasma.^49–53^ They established that protein resistance cannot be assigned to chemical identity alone but reflects the interfacial environment created by hydration, chain organization, steric accessibility, and electrostatic interactions. Data-driven approaches have subsequently extended these principles from qualitative design rules toward quantitative prediction. Early quantitative structure–property relationships (QSPR) predicted lysozyme and fibrinogen adsorption from molecular descriptors and assay variables,^54^ followed by neural-network models linking monolayer structure to wettability and protein adsorption.^55^ Subsequent studies incorporated brush thickness, molecular weight, grafting-related parameters, surface charge, and protein properties and, in some cases, used model predictions to guide experimental selection of new coatings.^56–59^ More recently, molecular dynamics (MD) simulations coupled with unsupervised learning have resolved hydration and ionic states within charged brushes,^60,61^ and representation-learning approaches have integrated chemical embeddings with brush and protein descriptors.^59^ These developments illustrate a progression from chemical heuristics toward increasingly quantitative and multiscale descriptions of polymer interfaces. However, these approaches also expose a persistent limitation—increasingly expressive algorithms cannot recover grafting heterogeneity, wet-state density profiles, ion partitioning, or aging histories when these variables were never measured. The field has advanced faster in model capacity than in defining the physical object that the model is expected to learn.

We use interfacial intelligence as an organizing framework for polymer-brush design (Figure 1). Here, interfacial intelligence refers to the capacity of an integrated model–experimental system to represent, predict, test, and iteratively update the physicochemical state realized by a polymer interface under defined operating conditions. Its central premise is that the relevant design target is not a nominal composition or an isolated structural descriptor but the realized interfacial state that connects molecular design to function. Operationally, such a framework should be state-resolved, condition-resolved, physics-grounded, experimentally testable, and experimentally updateable. State-resolved representations distinguish nominal descriptors from effective conformation, hydration, and ion organization. Condition-resolved representations define the solvent, pH, ionic strength, temperature, geometry, flow, and exposure history under which a state exists. Physics should constrain admissible states and model extrapolation rather than serve only as post hoc interpretation. Finally, mechanistic interpretation requires a testable prediction of how a defined perturbation changes the interfacial state and, consequently, function, with new measurements used to update the model.

**Figure 1:**
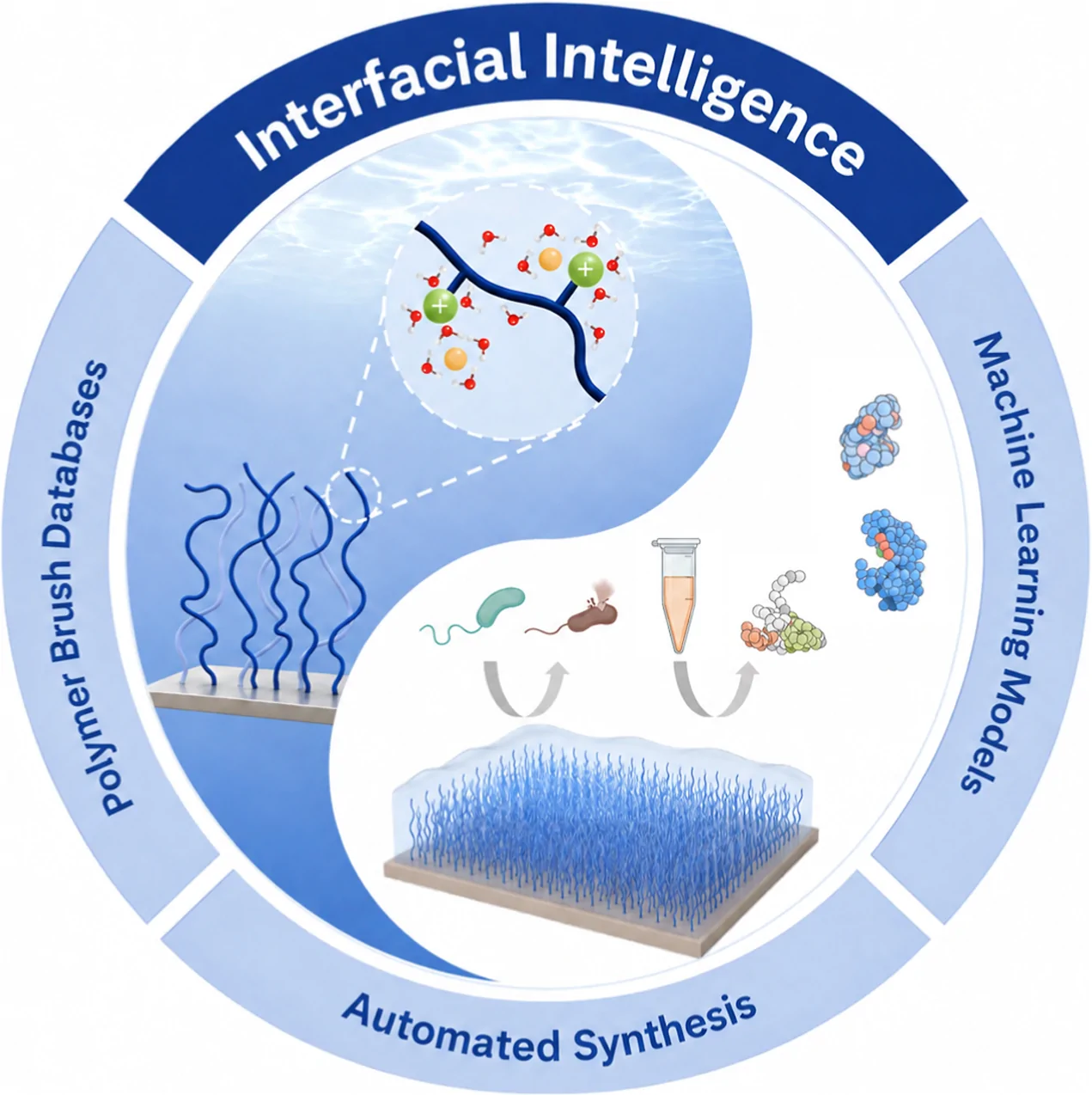
Conceptual framework of interfacial intelligence for polymer-brush design. Polymer-brush databases, machine learning models, and automated synthesis are integrated in a closed-loop workflow that connects molecular and structural design with the physicochemical interfacial state realized under operating conditions.

Several recent perspectives and reviews establish the foundations from which this perspective proceeds. Feng and Huang reviewed synthetic strategies and applications of polymer brushes.^62^ Pester, Klok, and Benetti examined synthesis, structural parameters, characterization challenges, and scalability.^63^ Masuda and Takai reviewed the use of controlled radical polymerization to construct well-defined soft biointerfaces, including antifouling polymer-brush surfaces.^64^ Conrad and Robertson highlighted molecular-weight distribution as a determinant of brush structure and function.^4^ Tang and co-workers surveyed machine learning approaches for antifouling surfaces.^65^ This Perspective focuses on the unresolved intersection of these areas. Using protein-resistant brushes as a recurring test case, complemented by examples from transport, lubrication, and polymer-grafted particle assembly, we examine how molecular and structural design can be connected to measurable interfacial states, how physical models and simulation can constrain data-driven learning, and how prospective experimentation can ultimately connect prediction back to synthesis and measurement. The central opportunity of interfacial intelligence is to move beyond direct composition–performance prediction toward a design-realized interfacial state–function relationship, in which predictive accuracy is coupled with physical interpretability, experimental accountability, and transferability across chemical and environmental space.

## 2 Representing Polymer Brushes for Data-Driven Learning

Physics-grounded learning starts with how polymer brushes are represented. Their behavior cannot be described by polymer chemistry alone. A useful representation should distinguish four connected levels: (1) molecular identity, (2) brush structure, (3) realized interfacial state, and (4) application performance (Figure 2). Molecular and structural variables describe what is synthesized. State variables describe what the interface becomes under the operating conditions. Performance variables define what is measured or is optimized. Machine learning can connect these levels, but the physical meaning of those relationships depends on how well each level is represented.^66^

**Figure 2:**
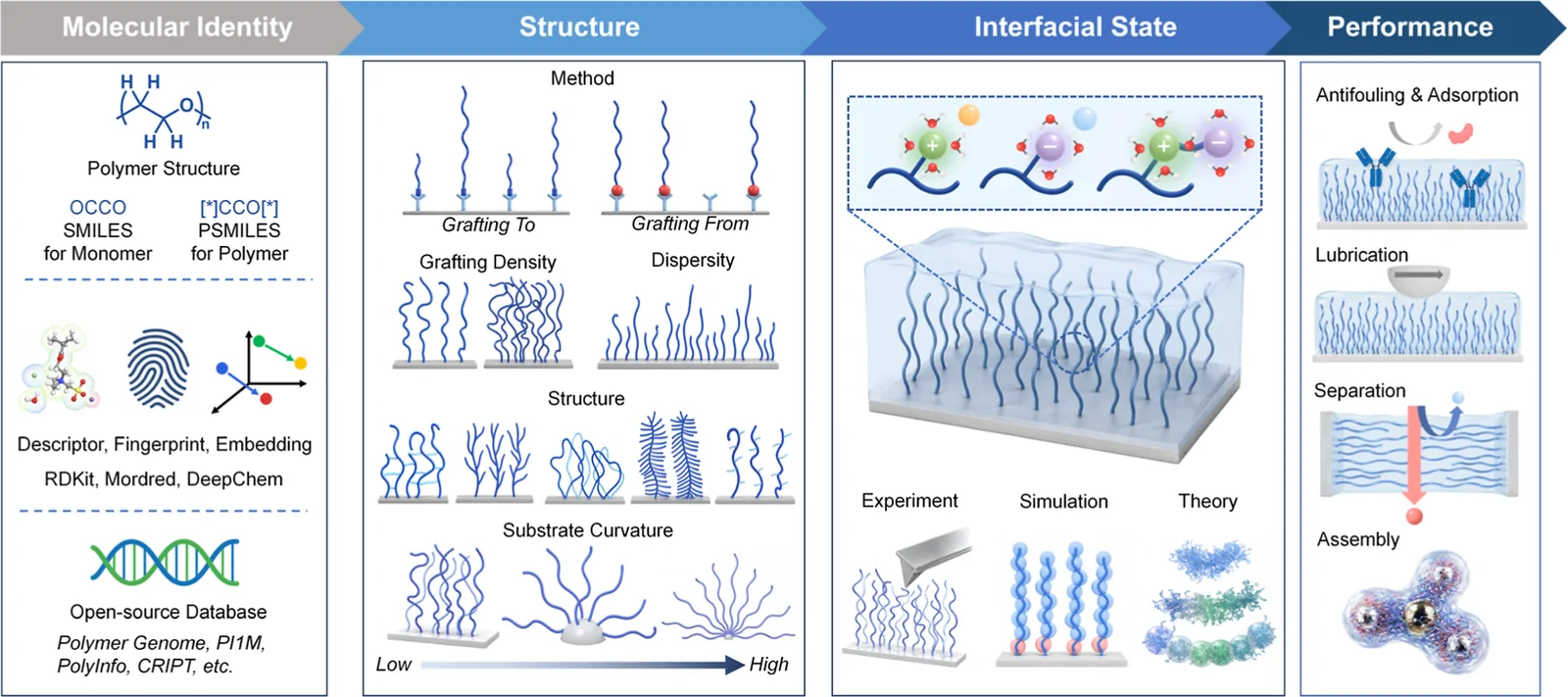
Multilevel representation of polymer brushes for data-driven and physics-grounded learning. Polymer-brush information is organized into four interconnected levels: molecular identity, brush structure, realized interfacial state, and functional performance. Molecular identity can be encoded using polymer representations, molecular descriptors, fingerprints, embeddings, and open-source databases. Brush structure is described by synthesis method, grafting density, dispersity, chain architecture, and substrate curvature. The resulting interfacial state, including chain conformation, hydration, ion organization, and local interactions, can be resolved through experiment, molecular simulation, and theory. These state variables ultimately govern application-relevant performance, including antifouling and adsorption, lubrication, separation, and assembly.

### 2.1 Molecular Identity of the Brush

The molecular identity provides the chemical layer of a machine-readable polymer-brush representation. Repeat-unit chemistry captures key interaction motifs, including hydration, ionization, and hydrogen bonding, and therefore provides substantial predictive information. Polymer SMILES (P-SMILES or PSMILES) provides a compact repeat-unit representation with defined connection points, whereas BigSMILES extends this framework to describe stochastic repeat-unit connectivity in random, block, and branched polymers, although neither fully captures the experimentally realized polymer ensemble.^67–70^ Resources such as PoLyInfo,^71,72^ Polymer Genome,^73^ PI1M,^67^ and CRIPT^74^ supply polymer structures, properties, processing metadata, reusable data schemas, or property-prediction informatics platforms, while RDKit,^200^ Mordred,^75^ and DeepChem^76^ can transform molecular structures into physicochemical descriptors, fingerprints, and graph representations.

Polymer brush studies demonstrate that this molecular layer contains substantial predictive information. Existing studies show that molecular representations provide substantial predictive information for brush wettability and protein adsorption, particularly when chemical descriptors or learned molecular embeddings are combined with brush-specific and interfacial variables.^77,78^ These results establish molecular identity as an essential representation layer while also showing that chemical encoding alone cannot specify the physicochemical state of a tethered interface.

### 2.2 Brush-Specific Structural Descriptors

A second representation layer describes the structural characteristics that distinguish a polymer brush from those of the corresponding untethered polymers. Relevant variables include grafting methods, chain architecture, molecular-weight distribution, grafting density, substrate geometry, and processing history. These descriptors distinguish, for example, a uniform linear brush from a branched or bimodal layer, a planar brush from a polymer-grafted nanoparticle, and interfaces produced through different grafting routes. Their relevance to data-driven learning arises from their control over chain crowding, segment accessibility, spatial organization, and ultimately the physicochemical state presented by the interface.

Existing machine learning studies demonstrate why multiple structural variables should be retained rather than collapsed into a single nominal descriptor.^58,79^ They further demonstrate that brush thickness or a single overlap parameter cannot fully represent the tethered-chain structure because chain length and lateral crowding may contribute independently. Molecular weight, grafting density, molecular-weight distribution, architecture, grafting method, and substrate geometry should therefore be retained as distinct but coupled structural variables. Their relevance lies not in serving as universal predictors but in determining the range of interfacial states accessible to the brush.

Experimental and simulation studies reveal additional structural variables that remain sparsely represented in current data sets. Gautham and Patra trained a deep-learning potential of mean force for single-chain-grafted nanoparticles using particle separation and the orientation of the grafting site relative to the interparticle axis.^80^ The learned interaction potential reproduced concentration-dependent assembly into anisotropic structures, including bilayers and percolated networks, showing that grafting geometry can carry predictive information that is absent from conventional planar-brush descriptors. Wang and co-workers’ experimental comparisons of poly­[*N*-(2-hydroxypropyl)­methacrylamide] (PHPMA) brushes prepared by grafting-to and grafting-from provide a complementary example.^34^ Grafting-from layers resisted blood-plasma fouling by approximately 1 order of magnitude more effectively despite polymers of comparable molecular weight because the two grafting methods produced different grafting densities and chain conformations. Future databases should therefore encode geometry, architecture, molecular-weight distribution, grafting statistics, and grafting method as distinct variables rather than treating them as implicit synthesis details.^81^

### 2.3 Static and Dynamic Interfacial State across Scales

The third representation layer describes the physicochemical state realized by a brush under operating conditions. Unlike molecular identity and nominal structural descriptors, these state variables may change without altering the underlying polymer chemistry and can evolve with solvent composition, ionic strength, pH, temperature, applied potential, mechanical loading, or exposure history.^35,82–86^ Relevant states span conformation, hydration, ion organization, mechanics, and molecular transport, from local molecular coordination and solvent dynamics to brush-scale swelling, relaxation, and adsorption.^60,87–90^ Information transfer across representation levels is hierarchical: molecular identity and brush structure define accessible states, operating conditions determine the realized interfacial state, and state-resolved descriptors connect this state to function. Useful intermediate descriptors include swelling, water content, segment profiles, ion uptake, mechanical response, and adsorption or transport kinetics. Their inclusion is central to physics-grounded learning because they provide the physical variables through which molecular and structural design can be connected to function.

Experimental techniques provide complementary observations of these states. Ellipsometry, X-ray reflectivity, and neutron reflectometry provide thickness-, swelling-, and depth-dependent composition.^35,91,92^ Atomic force microscopy (AFM) and surface-force measurements probe morphology, compression, and interaction forces.^93,94^ Quartz crystal microbalance with dissipation (QCM-*D*) and surface plasmon resonance (SPR) resolve solvent-coupled mass uptake and adsorption kinetics.^90,95^ Electrochemical impedance spectroscopy and electrokinetic measurements report charge-sensitive transport and interfacial electrical response.^89,96,97^ X-ray photoelectron spectroscopy and Fourier transform infrared (FTIR) spectroscopy provide information on composition, ionization, and local bonding environments.^98,99^ Soft X-ray absorption spectroscopy and X-ray emission spectroscopy provide complementary molecular-scale information on the local electronic and hydrogen bonding structure of water confined within hydrated polyelectrolyte brushes.^100^ None of these measurements alone provide a complete description of the interface, and many require physical models to convert raw signals into state variables. Their value for data-driven learning therefore lies not simply in adding more features but in providing experimentally grounded observation of the states that mediate interfacial function.

Current polymer-brush machine learning studies generally rely on reduced observables or proxies, such as thickness, contact angle, and ζ potential, rather than direct descriptions of the interfacial state.^57,78^ Although these variables improve prediction, they do not directly resolve hydration, ion organization, molecular accessibility, or internal density profiles. More state-resolved measurements are therefore needed to connect nominal brush descriptors with the physical environment experienced by an adsorbate.

Dai and co-workers combined QCM-D and SPR measurements across poly­(*N*-isopropylacrylamide) (PNIPAM), PEG, poly­(2-methacryloyloxyethyl phosphorylcholine) (PMPC), poly­(2-hydroxyethyl methacrylate) (PHEMA), and fluorinated brushes to establish a quantitative negative correlation between brush-associated water content and protein adsorption.^90^ Nagasawa and co-workers used sum-frequency generation spectroscopy together with QCM to show that the organization and interaction strength of interfacial water, rather than wettability alone, influence protein resistance.^37^ Murdoch and co-workers combined neutron reflectometry, AFM, and QCM-D to resolve ion-specific changes in the internal density profile and vertical phase separation of PNIPAM brushes near the lower critical solution temperature.^35^ Lin and Luo further connected ionic strength and applied potential with hydration, ion uptake, Young’s modulus, and protein binding in zwitterionic PMPC and poly­(sulfobetaine methacrylate) (PSBMA) brushes using AFM and electrochemical QCM-*D*.^36^ Although these studies were not designed as machine learning data sets, they identify experimentally accessible variables, including water content, depth-dependent grafting density, ion uptake, modulus, and adsorption kinetics, that could provide a more physical basis for future data-driven models. State-resolved data sets should therefore preserve both the measured observables and the assumptions or physical models used to infer state variables from them.

Molecular simulation combined with machine learning provides a complementary route to resolving microscopic interfacial states that are difficult to access directly by experiment.^101^ Ishraaq, Akash, and Das applied unsupervised clustering to all-atom MD trajectories of poly­[2-(methacryloyloxy)­ethyl trimethylammonium chloride] (PMETAC) brushes using local configurations of water, trimethylammonium groups, and chloride ions.^60^ The analysis resolved two hydration states of the same cationic functional group and showed that their populations varied with grafting density and counterion crowding. Bera and co-workers similarly clustered distance-based representations of water–water configurations within poly­(acrylic acid) (PAA) brushes of different charge fractions and counterions.^61^ Highly charged brushes exhibited the loss of several clusters associated with longer-range water organization, indicating that brush charge and steric confinement reorganize the water network rather than simply altering the total number of hydrogen bonds. These studies illustrate how unsupervised learning can reduce high-dimensional molecular trajectories into candidate microscopic state variables, including local coordination environments, hydrogen-bond configurations, and state populations. Such simulation-derived states should not, however, be treated as experimentally established mechanisms by themselves. Their physical significance can be strengthened by connecting predicted state populations and transitions to experimentally accessible observables such as vibrational spectra, scattering profiles, dielectric response, ion uptake, or adsorption kinetics.

Theory provides a complementary source of physical structure rather than another data-driven representation. Alexander–de Gennes scaling and self-consistent-field theory relate chain length, grafting density, and solvent quality to brush height, segment–density profiles, and compression, while polyelectrolyte-brush theories define regimes governed by counterion confinement, electrostatic screening, and charge regulation. Self-consistent field calculations have quantitatively reproduced brush compression and have been combined with AFM imaging to interpret lateral phase separation in mixed polystyrene/poly­(methyl methacrylate) (PS/PMMA) brushes, including the effects of grafting disorder and polydispersity on domain spacing.^102^ Direct examples integrating such theories, experimental brush data, and machine learning remain rare. In an interfacial-intelligence framework, theory should therefore enter before prediction rather than only after it. Physical models can constrain the admissible state space, define meaningful descriptors and extrapolation limits, and generate perturbation–response relationships that can be tested experimentally. Machine learning then operates within, or deliberately challenges, this physically defined domain.

### 2.4 Application-Relevant Performance and Learning Tasks

The final layer defines the functional target of learning, rather than the material representation itself. Performance should be described using metrics specific to the intended application and measured under explicitly defined operating conditions rather than being treated as a generic response variable.^58,103,104^ This distinction is important because the same brush may exhibit different apparent performance depending on the adsorbate, solvent, ionic environment, geometry, loading history, or measurement protocol.^78,103,105^ A useful learning target should therefore specify not only what is measured but also the conditions and protocol under which the measurement acquires physical meaning.

Antifouling currently provides the clearest examples of application-level targets in polymer-brush machine learning. Existing studies have predicted serum or single-protein adsorption from a combination of polymer chemistry, brush thickness, grafting density, wettability, ζ potential, and protein descriptors, with adsorbed mass or surface coverage serving as target variables.^57,58,78^ These studies establish protein adsorption as a tractable supervised-learning task, but they also illustrate why performance values should remain linked to experimental context. Adsorption measured for different proteins, concentrations, buffers, exposure times, and assay formats does not necessarily represent the same interfacial process. Application-level data sets should therefore preserve these conditions rather than treating adsorption as a context-independent material property. Other polymer-brush applications illustrate different forms of learning targets. For polymer-grafted nanoparticle assembly, deep-learning analysis of transmission electron microscopy (TEM) images has been used to quantify particle positions, sizes, and assembly states, extracting structural information that is difficult to obtain from ensemble-averaged characterization alone.^106^ Here, machine learning serves primarily as an analysis tool that converts image data into quantitative structural outputs rather than predicting performance directly. More recent active-learning simulations of binary polymer-grafted nanoparticle lattices connected grafting parameters, particle size, and stoichiometry with mechanical targets including modulus and toughness and associated the superior performance of NaCl-type assemblies with efficient packing and chain entanglement.^107^ These examples illustrate that learning tasks can range from extracting structural states from experimental data to predicting or optimizing application-level properties. Machine learning data sets remain much less developed for lubrication, membrane separation, and sensing, although experimental studies identify relevant performance variables and their dependence on brush state. For lubrication, appropriate targets include friction coefficient, load dependence, and cycling durability, which can vary with hydration, chain conformation, and ionic conditions. For membrane separations, permeability, selectivity, fouling resistance, and stability provide application-relevant targets and depend on grafting density, swelling, charge, and solvent or electrolyte environment. For sensing, relevant outputs may include sensitivity, selectivity, response time, recovery, and cycling stability. These experimental results do not yet constitute mature machine learning data sets, but they define the quantities that future data-driven studies should seek to predict and optimize.^103,108,109^ Future databases should retain application metrics together with operating conditions, test protocols, normalization methods, comparators, and data provenance. Such structured records preserve the link between brush design, interfacial state, and functional performance, improving data reliability, machine readability, and transferability across materials and environments.

## 3 From Predictive Models to Interfacial Intelligence

Once molecular identity, brush structure, interfacial state, and performance are represented separately, the next question is, what can be learned from their relationships? The key progression is therefore not from simple to increasingly complex algorithms but from end point prediction toward state resolution, physically constrained interpretation, and experimentally testable hypotheses. Regression identifies conditional relationships among the defined variables. High-dimensional analysis can reveal microscopic states or interaction patterns that are difficult to specify in advance. Molecular representation models describe chemical space, while literature-based tools organize the distributed experimental evidence. Active learning then connects predictions back to experiments by selecting measurements that refine the model or test a physical hypothesis.

### 3.1 Regression Models Identify Conditional Physicochemical Relationships

Regression models are well-suited to current polymer-brush data sets, which usually contain tens to hundreds of observations and combine molecular, structural, interfacial, environmental, and adsorbate variables. Their value extends beyond end point prediction. Comparison across related systems can reveal nonlinear trends, descriptor interactions, and chemistry-dependent responses that are difficult to identify from individual experiments. Tree-based and kernel models are particularly useful, because they capture such nonlinear relationships without assuming a predefined functional relationship.

Palai and co-workers illustrate this point by comparing serum-protein adsorption across poly­[*N*-(2-hydroxypropyl)­methacrylamide] (PHPMA), poly­(carboxybetaine methacrylamide) (PCBMA), poly­(ethylene glycol) (PEG), PHEMA, and poly­[2-(dimethylamino)­ethyl methacrylate] (PDMAEMA) brushes.^57^ Random forest regression (RF) outperformed support-vector regression (SVR), least absolute shrinkage and selection operator regression (LASSO), and gradient-boosting regression (GBR), with monomer hydrophobicity, film thickness and density, C–H bond count, and monomer charge among the important variables. More importantly, brush thickness showed opposite relationships with adsorption for different chemistries. Increasing thickness promoted serum adsorption for PDMAEMA and PHEMA but reduced it for PHPMA, PEG, and PCBMA. Thickness therefore does not define a universal antifouling rule. Depending on polymer chemistry, a thicker layer may increase the protein-accessible material or strengthen steric and hydration barriers.

Su, Masuda, and Takai reached a related conclusion for brush wettability.^77^ They compared linear, tree-based, kernel, nearest-neighbor, and multilayer-perceptron models for predicting the static water contact angle of chemically diverse brushes. Among the tested models, SVR gave the best prediction of static water contact angle, with a test *R* ^2^ of 0.86 and a root-mean-square error (RMSE) of 10.3°. SHAP analysis identified Mol log *P*, which represents molecular hydrophobicity, and PEOE_VSA7, which measures van der Waals surface area associated with weakly polar atomic environments, as positive contributors to water contact angle. Brush thickness provided additional structural information but did not replace the chemistry-derived variables. Molecular descriptors therefore captured the interaction propensity of the repeat unit, whereas thickness reflected how that chemistry was presented in the tethered layer. The combined contribution also explains why wettability cannot be assigned to repeat-unit chemistry alone.

Descriptor choice becomes especially important when structural variables are physically coupled. Okuyama, Sugawara, and Yamaguchi compared dry thickness, the chain-overlap parameter (*s*/2*R*_F_), and separate molecular weight and grafting density inputs for predicting protein adsorption on zwitterionic brushes (Figure 3a–c).^58^ Dry thickness and *s*/2*R*_F_ are physically meaningful descriptors, but both combine contributions from chain length and lateral crowding. Dry thickness (*h*) is coupled to molecular weight (*M*_n_) and grafting density (σ) through *M*_n_ = *h*ρ*N*_A_/σ, where ρ is the dry polymer density and *N*_A_ is Avogadro’s constant. The parameter *s*/2*R*_F_ describes the overlap between neighboring grafted chains by comparing the mean tether spacing (*s* ∝ σ^–1/2^) with the Flory radius of the hydrated chain (*R*_F_ = *lN* ^3/5^), where *l* and *N* are the monomer length and degree of polymerization, respectively. Values above and below unity broadly correspond to mushroom-like and overlapping brush regimes. These descriptors can therefore obscure the separate effects of chain length and lateral crowding. Among the tested linear and ensemble models, RF gave the best predictive performance. The representation using molecular weight and grafting density was more informative than those based on dry thickness or the chain-overlap parameter. SHAP analysis further assigned grafting density an importance approximately two to three times that of molecular weight within the sampled domain. This result does not establish density as a universal control variable. Rather, it shows that separating physically coupled variables can reveal relationships that are hidden in the descriptors. The predicted design map also identified a practical boundary—polymer-chain densities above approximately 0.4 chains nm^–2^ were absent from the experimental data, consistent with the limited range of grafting densities accessible in these systems.

**Figure 3:**
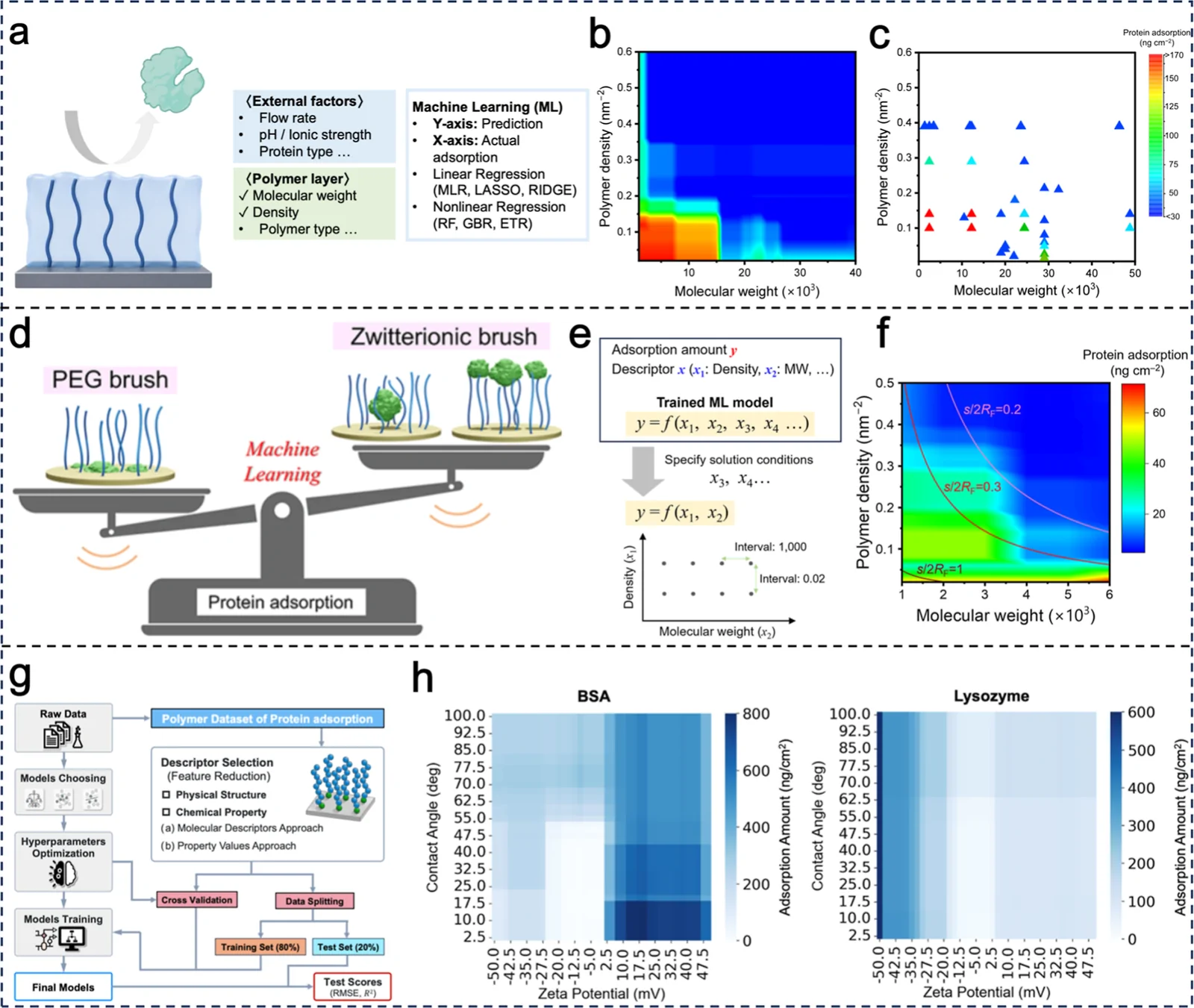
Machine learning analysis of structure–property relationships governing protein adsorption on polymer brushes. (a) Schematic of the descriptors and machine learning models used to predict protein adsorption. External factors, including flow rate, pH, ionic strength, and protein type, are combined with polymer-layer descriptors such as molecular weight, grafting density, and polymer type. Both linear regression models, including multiple linear regression and least absolute shrinkage and selection operator (LASSO), and ridge regression (RIDGE), and nonlinear models, including random forest (RF), GBR, and extra-trees regression (ETR), are considered. (b) Predicted protein-adsorption map as a function of molecular weight and grafting density for zwitterionic polymer brushes. (c) Experimental protein-adsorption data within the high-ionic-strength range of 100–200 mM. Reproduced with permission from ref 58. Copyright 2024, American Chemical Society. (d) Conceptual illustration of machine-learning-assisted comparison of protein adsorption on poly­(ethylene glycol) (PEG) and zwitterionic brushes. (e) Schematic procedure for generating predictive protein-adsorption maps from a trained machine learning model under specified solution conditions. (f) Predicted protein-adsorption map with boundaries of the chain-overlap parameter, *s*/2*R*_F_, indicating different grafted-chain conformational regimes. Reproduced with permission from ref 79. Copyright 2025, American Chemical Society. (g) Machine learning workflow for predicting protein adsorption on polymer-brush surfaces, including descriptor selection, data splitting, cross-validation, hyperparameter optimization, model training, and performance evaluation. (h) Two-dimensional protein-adsorption maps for bovine serum albumin (BSA) and lysozyme as functions of ζ potential and water contact angle, generated using the trained random-forest model. Reproduced with permission from ref 78. Copyright 2025, American Chemical Society.

The same structural variables can also acquire different meanings in different chemical environments. Okuyama, Sugawara, and Yamaguchi subsequently compared protein adsorption on PEG and zwitterionic brushes using the same set of structural and environmental descriptors. RF performed best for PEG brushes, whereas ETR gave the best performance for zwitterionic brushes (Figure 3d–f).^79^ More importantly, the two systems showed distinct adsorption landscapes with molecular weight and grafting density. For PEG brushes, molecular weight had a modest protective effect, while grafting density showed a nonmonotonic relationship with adsorption. Effective protein resistance required substantial chain overlap, with *s*/2*R*_F_ approaching or falling below 0.3 rather than simply crossing the conventional mushroom–brush boundary at *s*/2*R*_F_ = 1. For zwitterionic brushes, increasing molecular weight and grafting density was more consistently associated with reduced adsorption, while ionic strength introduced an additional dependence on the variables through charge screening and ion-dependent hydration.^47,48^ These different trends can be understood in the context of established protein-adsorption models for polymer brushes. For PEG brushes, primary adsorption at the underlying substrate remains important because chain fluctuations can permit protein penetration, particularly at lower chain overlap. In zwitterionic brushes, adsorption can also involve interactions at the brush periphery and within the hydrated layer. Changes in molecular weight or grafting density therefore do not have equivalent effects in the two systems. Their influence depends on how brush structure regulates substrate accessibility, protein penetration, electrostatic interactions, and hydration.

The relationship also depends on the adsorbate. Su, Masuda, and Takai showed that ζ potential affected negatively charged bovine serum albumin and positively charged lysozyme in opposite directions, whereas hydrophobicity became more important near electrical neutrality (Figure 3g,h).^78^ Thus, even a measured interfacial property such as ζ potential cannot be interpreted independently of the molecule interacting with the brush.

These studies show that regression can connect the three classes of information. Molecular descriptors describe the interaction propensity of the polymer, brush descriptors capture chain organization and steric accessibility, and interfacial descriptors describe the environment encountered by a particular adsorbate. The relationships among these variables are often nonlinear and depend on the chemistry, brush structure, operating conditions, and adsorbate identity. Regression models are useful for identifying such interactions, regime boundaries, and reversals, but feature importance indicates predictive association within the sampled domain rather than causation. These examples therefore illustrate a central criterion of interfacial intelligence: a useful descriptor is not one that is merely predictive but one whose relationship with function can be traced to a condition-dependent interfacial state and tested by controlled perturbation.

### 3.2 High-Dimensional Learning Helps Resolve Interfacial States and Interaction Patterns

Regression is most informative when relevant descriptors can be defined in advance. This becomes more difficult for molecular trajectories, images, and other high-dimensional data where the important physical coordinates may not be obvious. Learning methods can help reduce these data to a smaller set of states, interactions, or spatial features. Their main value is therefore not greater model complexity but the ability to identify physical information that is missing from conventional descriptors.

Early nonlinear models remained largely within predefined descriptor spaces. Kwaria and co-workers used an artificial neural network to predict water contact angle and fibrinogen adsorption on self-assembled monolayers from elemental composition and bond-type descriptors.^55^ Liu and co-workers combined random-forest feature selection with a descriptor-based artificial neural network for repurposing existing polymer brushes and a functional-group-based SVR model for designing new candidates (Figure 4a–c).^110^ Charge–charge interaction energy, mean first ionization potential, and the CH3× and CH2 × 2 atom-centered fragments were strongly associated with protein resistance, with probabilities of 98–100%. In contrast, rotatable-bond fraction, oxygen content, and ALOGP (Ghose-Crippen octanol–water partition coefficient) were associated with increased protein adsorption. The joint contribution of charge–charge energy and CH3× was consistent with zwitterionic motifs that support strong ionic hydration and impede protein approach. Experimental validation in undiluted serum and plasma then advanced the study from retrospective correlation to prospective material selection. These models broadened chemical screening, but their physical interpretation remained constrained by the descriptors chosen in advance. More recent approaches move closer to interfacial intelligence by learning hydration states, transport-active regions, effective interactions, and mesoscale structures directly from high-dimensional data.

**Figure 4:**
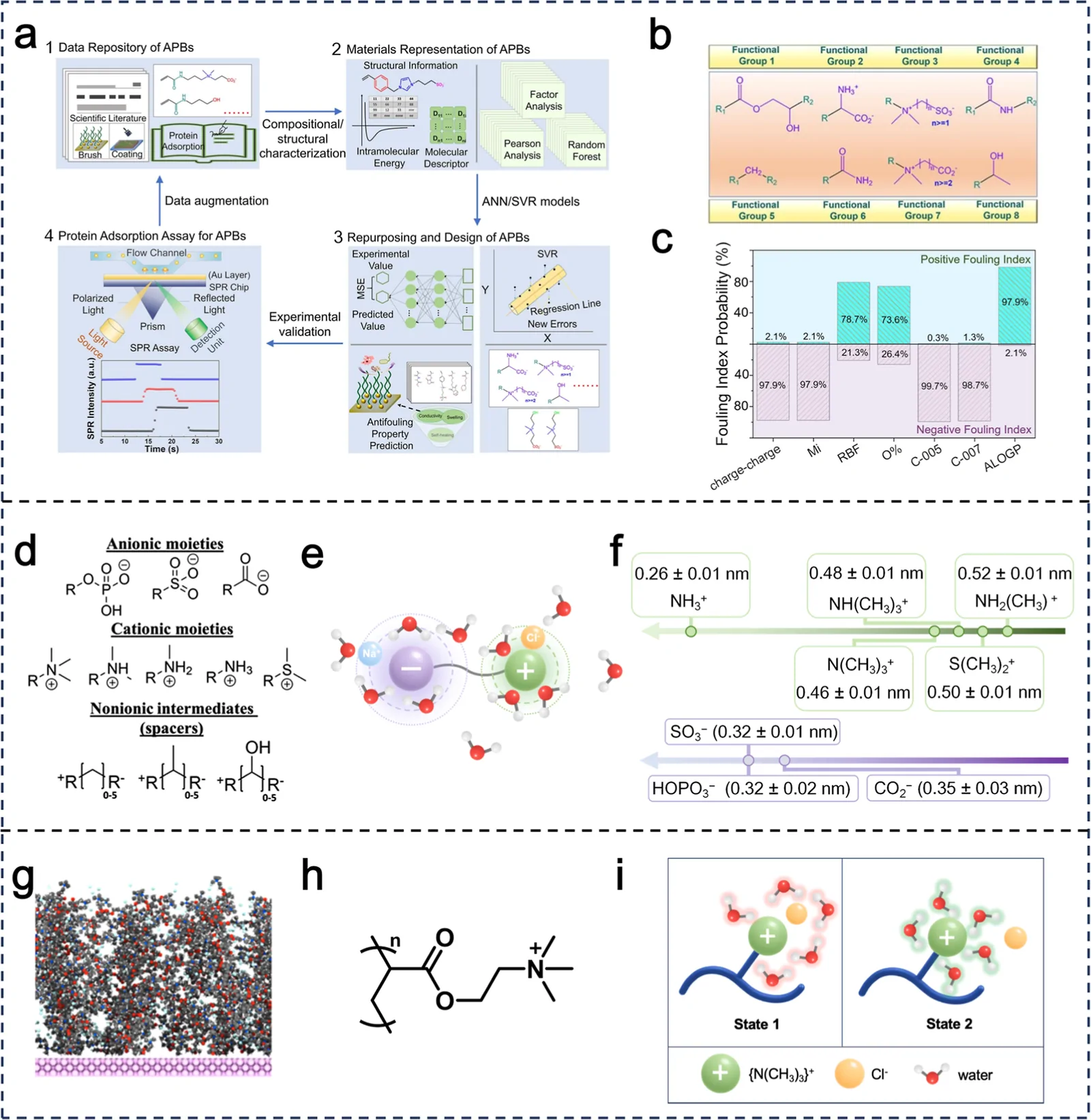
Machine-learning-enabled identification of molecular descriptors and hydration states relevant to antifouling polymer brushes. (a) Four-step machine learning workflow for the repurposing and design of antifouling polymer brushes, including data collection, materials representation, model construction, and experimental validation. (b) Representative functional groups used for antifouling polymer-brush design. (c) Predicted positive and negative fouling-index probabilities of seven molecular descriptors in the artificial neural network (ANN) model. Reproduced with permission from ref 110. Copyright 2021, Elsevier. (d) Representative anionic, cationic, and nonionic spacer moieties used to construct zwitterionic molecules. (e) Schematic illustration of water and counterions associated with charged zwitterionic moieties to form local coordination shells. (f) Comparison of hydration-shell radii for representative cationic and anionic moieties. Reproduced with permission from ref 111. Copyright 2023, Royal Society of Chemistry. (g) Representative molecular-dynamics snapshot of poly­[2-(methacryloyloxy)­ethyl trimethylammonium chloride] (PMETAC) brushes, where dark gray, blue, cyan, red, and pink particles represent carbon, nitrogen, chloride, oxygen, and wall atoms, respectively. (h) Chemical structure of PMETAC. (i) Schematic representation of two distinct hydration states around the trimethylammonium, N­(CH_3_)_3_^+^, group, illustrating differences in the organization of surrounding water and chloride ions. Reproduced with permission from ref 60. Copyright 2024, American Chemical Society.

Molecular simulation provides access to a richer set of physical variables. Christiansen, Cheng, and Mehraeen trained eXtreme Gradient Boosting (XGBoost) models on a molecular-dynamics library of 240 zwitterions constructed from different cationic, anionic, and spacer units (Figure 4d–f).^111^ Hydration was described by coordination-shell radius, coordination number, and effective water residence time around each charged moiety. The models showed that cation hydration depended strongly on steric accessibility and hydrogen bonding capacity. The hydration-shell radius around the zwitterionic cation followed the order: NH_3_^+^ 0.26 ± 0.01 nm < N­(CH_3_)_3_^+^ 0.46 ± 0.01 nm < NH­(CH_3_)_2_^+^ 0.48 ± 0.01 nm < S­(CH_3_)_2_^+^ 0.50 ± 0.01 nm < NH_2_(CH_3_)^+^ 0.52 ± 0.02 nm. Fully protonated amines formed a distinct hydration regime, whereas bulkier methylated cations generally supported larger and longer-lived hydration shells. The hydration-shell radius around the zwitterionic anion followed the order: HOPO_3_^–^ 0.32 ± 0.02 nm ∼ SO_3_^–^ 0.32 ± 0.01 nm < CO_2_^–^ 0.35 ± 0.03 nm. Anion hydration depended not only on the anionic group itself but also on the size and charge of the neighboring cation, indicating that the two local solvation environments were coupled. Carboxylate, sulfonate, and phosphate groups therefore did not behave as isolated hydration centers. Their hydration reflected the coupled electrostatic and steric field of the entire zwitterion. By contrast, ion-association properties were predicted much less accurately. Adding hydration descriptors produced only limited improvement. This contrast suggests that first-shell hydration is more directly encoded by a local molecular structure, whereas ion association depends on higher-order effects, such as spacer conformation, competing hydration, and ion-specific solvation.

While regression models rely on experimentally defined descriptors to establish structure–property relationships, representation-learning approaches provide a pathway to uncover latent interfacial states that are difficult to describe using conventional variables. Ishraaq, Akash, and Das used unsupervised learning of atomistic trajectories to resolve two hydration states around the same N­(CH_3_)_3_^+^ group in poly­(2-(methacryloyloxy)­ethyl trimethylammonium chloride (PMETAC) brushes (Figure 4g–i).^60^ State-1 contained less structured water with a lower tetrahedral-order parameter, whereas state-2 contained more tetrahedrally ordered water with a higher tetrahedral-order parameter. The states differed primarily in the tetrahedral order of the surrounding water, rather than in hydration number or average dipole orientation. The methyl-shielded quaternary ammonium group has a relatively low local charge density and supports an apolar, tetrahedrally ordered hydration environment. Increasing grafting density from 0.15 to 0.25 chains nm^–2^ did not appreciably change the geometry or separation of the two states but increased the state-1/state-2 population ratio from 24 to 44. Over the same range, the fraction of water shared between the N­(CH_3_)_3_^+^ and Cl^–^ hydration shells increased from 19% to 28%. Because Cl^–^-associated water has lower tetrahedral order, this hydration-shell overlap progressively suppressed the more structured state. The nearly invariant cation–anion distance and almost 100% screening efficiency ruled out stronger contact ion pairing or counterion expulsion. Thus, counterion crowding changed the hydration-state population by increasing cation–Cl^–^ hydration-shell overlap, rather than by shortening the cation–anion distance. Bera and co-workers extended this state-resolved analysis to anionic poly­(acrylic acid) (PAA) brushes with different charge fractions and counterions.^61^ Clustering of water–water configurations revealed that increasing ionization progressively disrupted long-range water-network motifs, reduced the configurational range of hydrogen-bond connectivity, and increased hydrogen-bond angles, indicating a less connected and more distorted hydrogen-bond network. By comparing uncharged, partially charged, and fully charged brushes, the study separated the effects of polymer confinement from electrostatic interactions and counterion-mediated solvation. The results showed that conventional bulk-water hydrogen-bond criteria overestimate hydrogen bonding inside charged brushes because water molecules simultaneously respond to polymer segments, carboxylate groups, and counterions. Importantly, counterion effects were not determined solely by valence. Highly hydrated monovalent ions strongly disrupted local water networks through their high population density, whereas multivalent ions induced distinct perturbations through stronger electrostatic coupling and ion-mediated bridging. Together with the PMETAC study, these results demonstrate that brush hydration cannot be reduced to a single descriptor such as charge density. It emerges from coupled functional-group chemistry, ion distribution, steric confinement, and water organization.

Beyond hydration-state identification, machine learning can also identify molecular events associated with dynamic brush functions. Ishraaq and Das combined all-atom MD simulations with linear discriminant analysis (LDA) to identify local molecular descriptors associated with nonlinear electro-osmotic transport in PMETAC-brush-grafted nanochannels.^112^ Instead of using predefined high-level descriptors such as hydration number or hydrogen-bond parameters, they constructed low-domain-knowledge descriptors based on local coordination statistics, including the number of chloride ions, water oxygen atoms, and neighboring monomers around selected brush atoms. LDA projected these high-dimensional interfacial configurations into a maximally separated low-dimensional space and ranked the discriminative importance of each local interaction descriptor, thereby identifying “what to study and where to study” within the brush layer. The analysis associated the enhanced electro-osmotic response with a coupled rearrangement of the brush–ion–water interface: electric-field-induced deformation of PMETAC chains localized counterions near the brush–bulk boundary, where hydrated ions could migrate more freely and carry associated water molecules, generating an unexpectedly large flow response. This study provides an important example of interpretable machine learning for prioritizing physically meaningful mechanistic hypotheses, where machine learning does not replace molecular simulation but reduces the enormous analysis space and converts complex trajectories into physically meaningful interfacial variables.

Deep learning also connects molecular-scale brush interactions to mesoscale assembly. Gautham and Patra developed a deep neural network-based potential of mean force (DL-PMF) model by learning polymer-grafted nanoparticle interaction energies directly from coarse-grained MD trajectories.^80^ The model employed Behler–Parrinello-type radial and angular symmetry functions to encode the local chemical environment around nanoparticle centroids and polymer grafting sites, enabling the prediction of orientation-dependent interparticle interactions. Unlike conventional distance-dependent PMFs, which neglect directional effects, the DL-PMF identified graft-site orientation as an important predictive descriptor within the simulated domain for anisotropic interactions, allowing accurate reproduction of complex assemblies including bilayer and percolating structures. Qu, Jimenez, and Kumar developed a deep-learning-based image analysis framework using a convolutional neural network (CNN, YOLOv3 object detection architecture) trained on a manually labeled nanoparticle TEM data set (DOPAD).^106^ By integrating sliding-window and image-merging algorithms, the model automatically extracted nanoparticle coordinates and sizes from large-scale TEM images, converting complex real-space morphologies into quantitative structural descriptors. The extracted particle distributions enabled direct analysis of assembly states, including dispersion, clustering, and anisotropic ordering, and provided a real-space complement to small-angle X-ray scattering/small-angle neutron scattering (SANS) measurements by linking microscopic morphology with quantitative structure factors. These studies mark a progression from predicting an end point to identifying hydration populations, ion distributions, transport-active regions, interaction anisotropy, and assembly morphology. Their mechanistic value nevertheless depends on validation against independent observables, because a learned cluster, embedding, or effective potential remains conditional on its representation, simulation model, and sampled state space.

### 3.3 Language Models Connect Molecular Representations with Distributed Evidence

Language models contribute to polymer-brush informatics in two distinct ways. Chemical language models learn molecular representations from polymer strings, whereas generative large language models (LLMs) organize scientific text and connect distributed information.^113^ BB-EIT provides a brush-specific example of the first role. Su, Tanaka, Ushiku, and Takahashi used a pretrained ChemBERTa encoder to convert monomer SMILES into 768-dimensional chemical embeddings (Figure 5a–d).^59^ These embeddings were combined with brush thickness, water contact angle, ζ-potential, protein isoelectric point, and protein molecular weight to predict adsorption of BSA, lysozyme, and fibrinogen. The original data set contained 101 measurements across nonionic, ionic, and zwitterionic brushes. SMILES enumeration and heteroscedastic label perturbation expanded the training set to 6400 entries, increasing the test *R* ^2^ from 0.73 to 0.88. Removing brush thickness, water contact angle, and ζ-potential reduced the test *R* ^2^ from 0.88 to 0.70. The decrease in predictive performance after removal of the interfacial descriptors provides a direct example of why molecular embeddings alone cannot uniquely specify the realized brush interface. The physical interpretation of BB-EIT also illustrates this distinction. Chemical embeddings represented the interaction repertoire of the repeat unit, whereas brush thickness, water contact angle, and ζ-potential provided information related to steric accessibility, wettability, and electrostatics. SHAP analysis ranked chemical embedding, protein charge, ζ-potential, and water contact angle among the important inputs. These associations support a picture in which protein adsorption depends on coupled chemical and interfacial factors rather than on a single antifouling motif. However, they do not resolve hydration structure, penetration depth, or adsorption free-energy barriers. The mechanistic interpretation is further limited because water contact angle and ζ-potential were imputed for part of the data set, and SMILES enumeration creates alternative strings rather than new physical states.

**Figure 5:**
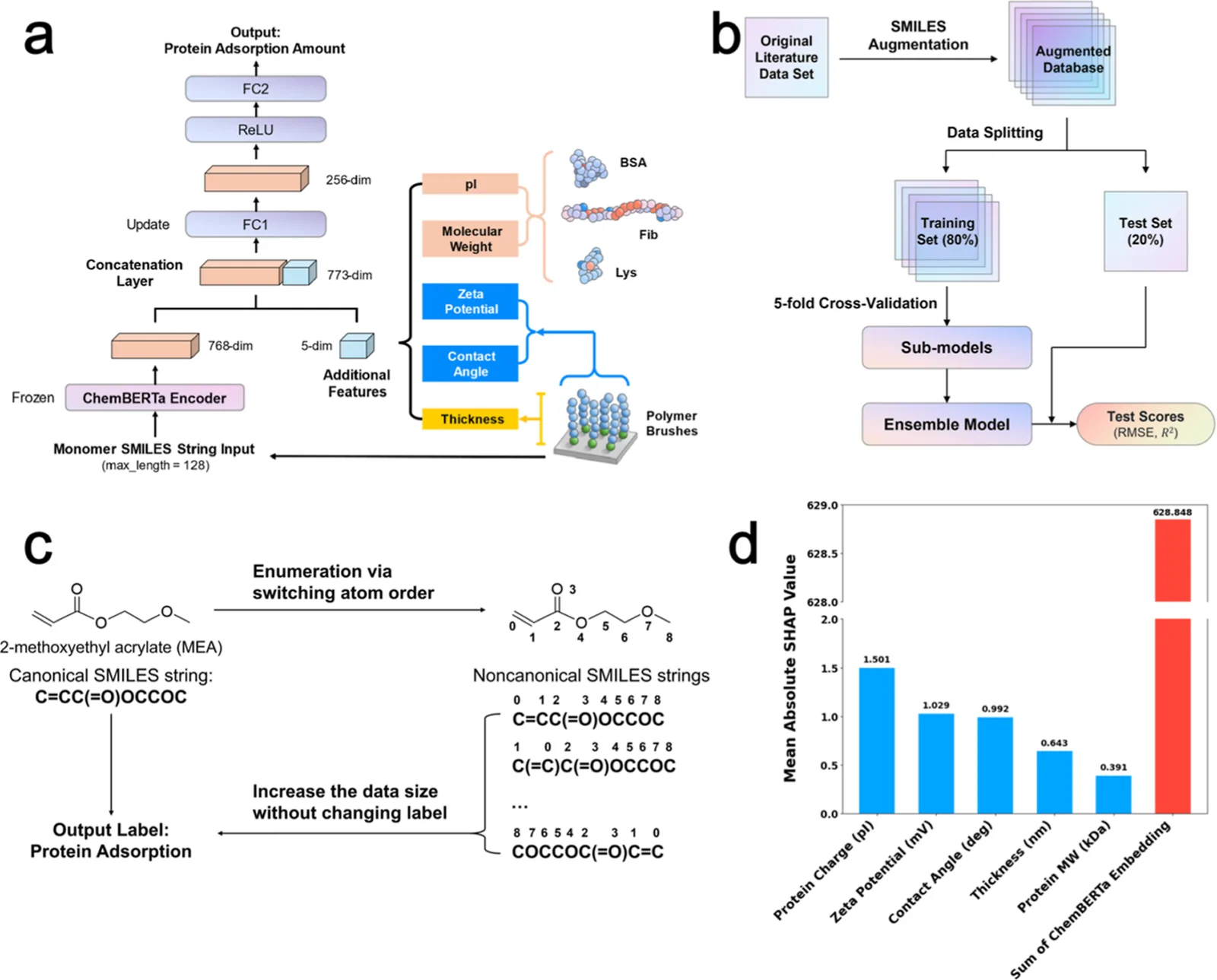
Chemical representation learning and data augmentation for protein-adsorption prediction on polymer brushes. (a) Architecture of BB-EIT (Biointerface BERT Encoder for Interaction Translation) for predicting protein adsorption on polymer brushes. (b) Schematic workflow of BB-EIT training and model evaluation. (c) Illustration of data augmentation through SMILES enumeration using 2-methoxyethyl acrylate (methoxyethyl acrylate) as an example. (d) Mean absolute SHAP values for the FC1 output of BB-EIT, indicating the relative contributions of chemical embeddings and additional descriptors. Reproduced with permission from ref 59. Copyright 2026, American Chemical Society.

Related polymer language models suggest how molecular representation could be expanded further. PolyBERT was pretrained on approximately 80 million polymer structures and generated compact fingerprints for multitask property prediction, showing that transformer encoders can learn chemical similarities beyond fixed fragment counts.^114^ PolyLLMem subsequently fused Llama 3 text embeddings with Uni-Mol structural embeddings and adapted both using low-rank fine-tuning, demonstrating that linguistic and three-dimensional molecular representations can provide complementary information in small polymer data sets.^115^ Uni-Poly similarly combined SMILES, molecular graphs, three-dimensional geometries, fingerprints, and generated textual descriptions into a multimodal polymer representation.^116^ Such representations could help distinguish subtle changes in ionic groups, spacer chemistry, comonomer identity, and anchoring functionality in polymer brushes. They still describe only a part of the interface. Grafting statistics, wet thickness, curvature, solvent composition, ion partitioning, and adsorbate properties must be represented separately if the model is to capture how molecular chemistry is expressed under the operating conditions.

Generative LLMs address a different bottleneck–polymer-brush data that are scattered across articles, figures, captions, and experimental sections. Existing literature-mining studies show the scale of this opportunity. MaterialsBERT extracted approximately 300,000 polymer-property records from about 130,000 abstracts. A later full-text workflow combining MaterialsBERT with GPT-3.5 processed approximately 681,000 polymer-related articles and produced more than one million records covering 24 properties of over 106,000 polymers.^117^ These systems increased the scale of data extraction, but complex experimental relationships still require filtering, postprocessing, and validation. This problem is especially important for polymer-brush research because the relevant information is relational. A useful record should connect polymer identity with the substrate, grafting method, molecular-weight distribution, grafting density, wet-state characterization, medium composition, adsorbate identity, measurement protocol, and time-dependent performance. The LLM should therefore populate a provenance-preserving data graph and flag missing or conflicting fields rather than compress heterogeneous experiments into apparently complete rows.

Tool-assisted agents extend this role from evidence organization to workflow execution. ToPolyAgent, for example, connects LLMs with coarse-grained simulation tools and can construct and run bead–spring models for linear, ring, star, dendritic, and brush architectures through natural-language instructions.^118^ Its contribution is operational rather than mechanistic: the agent translates a scientific request into topology construction, simulation setup, execution, and analysis, while the physical validity remains determined by the force field, boundary conditions, sampling, and user-defined model. Language models therefore serve mainly as interfaces between chemical representations, distributed evidence, and computational tools. They can improve how information is encoded, retrieved, and connected, but they do not establish physical validity on their own. A credible brush agent must cite every extracted datum, expose its assumptions, test predictions against competing mechanisms, and verify that the intended realized interfacial state was actually produced. To our knowledge, no peer-reviewed study has yet demonstrated such a fully validated, state-aware closed loop for polymer brushes.

### 3.4 Active Learning Connects Prediction with Experimental Decisions

Representation and prediction do not by themselves close the design loop. The next step is to decide which experiment or simulation should be performed next. Active learning and autonomous synthesis remain at an early stage for polymer brushes.^119^ To our knowledge, existing studies have demonstrated machine learning-guided candidate selection and improved experimental control over brush growth, but no peer-reviewed platform has yet closed the full predict–synthesize–characterize–update loop for polymer-brush interfaces using uncertainty-aware acquisition. A key brush-specific advance is continuous-flow surface-initiated photoinduced electron transfer–reversible addition–fragmentation chain-transfer polymerization (SI-PET-RAFT) developed by Kuzmyn and co-workers, which enabled oxygen- and water-tolerant, metal-free growth with substantially improved kinetic control (Figure 6a–e).^120^ Poly­(methoxy oligo­(ethylene glycol)­methacrylate) (PMeOEGMA) brushes grew linearly to ∼250 nm under flow, whereas static polymerization plateaued below 50 nm. The platform also enabled reinitiation and diblock-brush formation, while the linear relationship between dry thickness and molecular weight established thickness as a practical indicator of chain growth. The improvement arose mainly from continuous replenishment of the reaction medium rather than photocatalyst replacement alone. Under static conditions, growth plateaued at ∼36 nm after 90–120 min, whereas continuous flow sustained active species transport and delayed kinetic arrest. This study provides an important experimental foundation for adaptive brush synthesis. It also shows that reaction time alone is not sufficient to define the realized brush structure. Flow rate, irradiation, mass transport, initiator activity, and monomer supply jointly influence chain growth, molecular-weight distribution, and surface uniformity.

**Figure 6:**
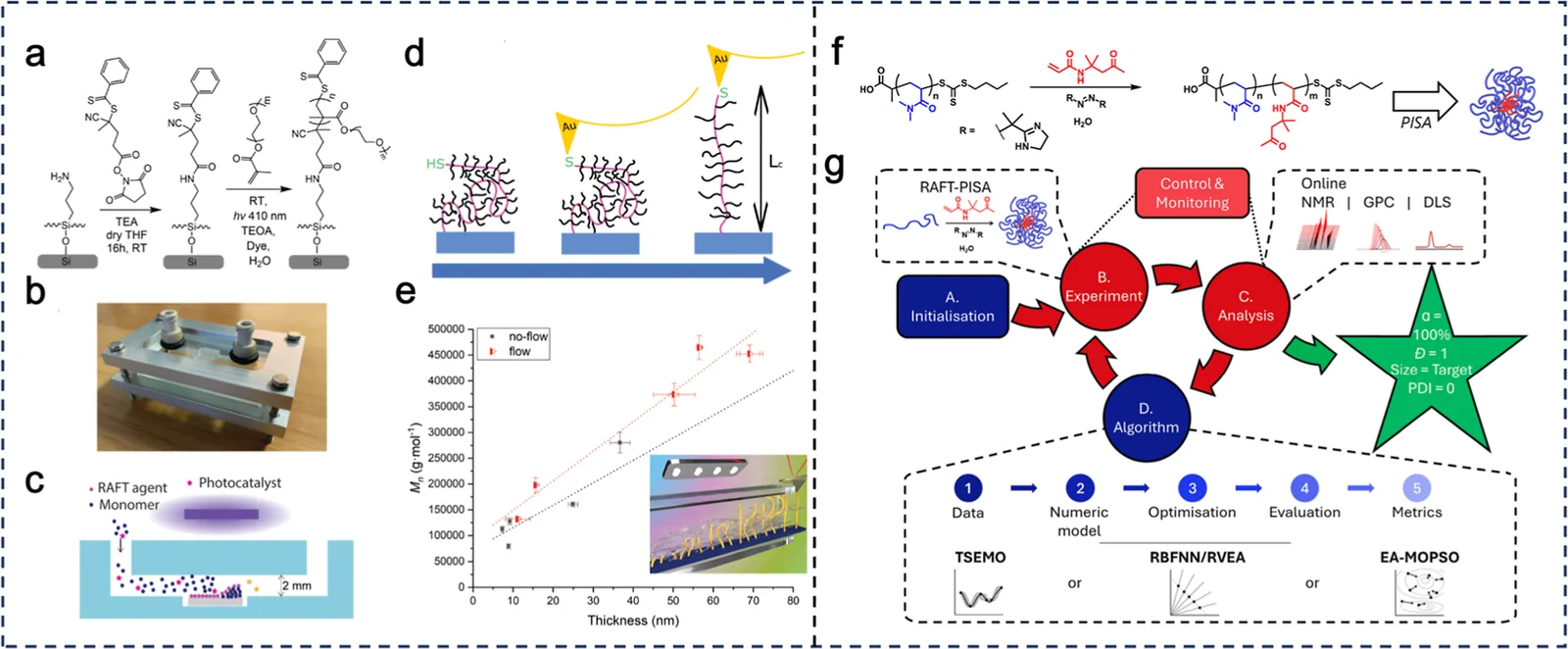
Continuous-flow and autonomous polymer synthesis for controlled polymer growth and materials optimization. (a) Chemical reactions involved in surface-initiated photoinduced electron transfer–reversible addition–fragmentation chain-transfer (SI-PET-RAFT) polymerization. (b) Photograph of the flow reactor. (c) Schematic illustration of SI–PET–RAFT under continuous-flow conditions. (d) Schematic of single-molecule force spectroscopy measurements of polymer brushes. (e) Relationship between dry brush thickness and molecular weight (*M*_n_) for polymer brushes prepared by SI-PET-RAFT under flow and no-flow conditions. Reproduced with permission from ref 120. Copyright 2023, Royal Society of Chemistry. (f) Reaction scheme of RAFT-mediated polymerization-induced self-assembly (RAFT-PISA). (g) Schematic workflow of a self-driving laboratory for polymer nanoparticle synthesis by RAFT dispersion polymerization, integrating automated experimentation, online nuclear magnetic resonance (NMR), gel permeation chromatography (GPC), dynamic light scattering (DLS) characterization, data analysis, and multiobjective optimization. Reproduced with permission from ref 124. Copyright 2025, Royal Society of Chemistry.

What remains missing in polymer-brush synthesis is the decision layer that determines how these variables should be changed from one experiment to the next. An early example in an adjacent polymer-design problem was reported by Tamasi and co-workers, who coupled active machine learning with robotic synthesis and characterization to explore random-copolymer compositions for stabilizing polymer–protein hybrids. Although not a polymer-brush system, this study illustrates how prospective model-guided experimentation can navigate a large polymer chemical space and iteratively prioritize new compositions for experimental testing.^121^ Autonomous RAFT platforms in adjacent polymer systems provide examples of how such a loop could operate. Grimm and co-workers combined a computer-controlled flow reactor with inline NMR spectroscopy, online GPC, and Thompson-sampling-based multiobjective optimization to balance conversion against dispersity.^122^ The same platform was subsequently used to control postpolymerization amidation by varying amine feed ratios, demonstrating automated regulation of both chain growth and chemical functionality. These studies show how controlled synthesis, rapid characterization, and sequential decision-making can be integrated within a common experimental workflow.^123^ Physical knowledge can further improve the efficiency of this search. Wilding and co-workers incorporated mechanistic priors into Bayesian optimization of RAFT polymerization. The resulting platform identified the conversion–dispersity Pareto front more efficiently than a purely experimental workflow. It achieved a larger hypervolume after 15 experiments than the fully experimental campaign reached after 22 and directed the search toward lower-temperature conditions that suppressed termination and produced narrower molar-weight distributions.

A more complex self-driving platform for reversible addition–fragmentation chain-transfer polymerization-induced self-assembly (RAFT-PISA) illustrates why polymer-brush optimization often involves several objectives at once. Knox and co-workers integrated flow synthesis with NMR, GPC, and DLS to optimize conversion, dispersity, particle size, and particle-size dispersity simultaneously (Figure 6f,g).^124^ A 67-experiment campaign was completed in 4 days with minimal user intervention and produced particles ranging from 34 to 116 nm. This study showed that conversion was controlled mainly by temperature, dispersity increased with target chain length, and particle size depended primarily on the degree of polymerization of the hydrophobic block. Three optimization strategies (i.e., TSEMO, RBFNN/RVEA, and EA-MOPSO) displayed different exploration–exploitation behavior. The acquisition strategy therefore influenced not only the optimum identified, but also which region of material space became experimentally characterized.

These adjacent polymer platforms define the decision framework that could be transferred to polymer brushes. A brush-specific closed loop should connect three levels—synthesis, interfacial state, and function. Synthesis conditions determine chain length, grafting density, molecular-weight distribution, and structural uniformity. State-resolved characterization then establishes what interface was actually formed, including hydration, chain conformation, ion organization, or environmental response. Functional measurements report the resulting antifouling, frictional, transport, or other application performance. Rather than pursuing the best end point alone, active learning should select experiments that distinguish whether performance changes arise from altered brush architecture or interfacial hydration. Automation then executes the experiment, while state-resolved characterization converts optimization into transferable physical insight.

## 4 Summary and Outlook: Toward Interfacial Intelligence for Polymer Brushes

Polymer-brush informatics is beginning to move beyond simple composition–property relationships. The studies discussed in this Perspective show that molecular chemistry, brush structure, operating conditions, and adsorbate identity can have strongly coupled effects on interfacial function. PEG and zwitterionic brushes, for example, respond differently to similar changes in chain packing because steric accessibility, hydration, and electrostatic interactions contribute differently in nonionic and ionic layers.^79,125,126^ Simulations of PMETAC and PAA brushes further show that charge, grafting density, and counterion identity reorganize confined water in ways that are not captured by average hydration alone.^60,61,127^ In polymer-grafted nanoparticle systems, graft-site orientation introduces directional interactions that alter assembly pathways.^80,128^ Together, these studies illustrate the value of data-driven approaches for identifying condition-dependent relationships beyond composition-only descriptions, while also highlighting that the strength of mechanistic evidence can vary substantially. Feature importance quantifies the contribution of descriptors to model predictions within the sampled domain but does not by itself establish causality. Clustering separates configurations according to a chosen representation.^60^ Learned potentials reproduce interactions within the simulated state space.^80^ None of these results alone establishes mechanism. Stronger evidence requires a controlled perturbation of the proposed physical variable followed by the predicted change in state and function. Examples include a grafting-density-induced shift in water organization, an ion-specific change in hydration-state populations, or an orientation-dependent transition in nanoparticle assembly.^129–134^ A useful model should therefore do more than identify a correlated descriptor. It should specify which interfacial state is expected to change, under what conditions, and what measurable response should follow.

Achieving this goal first requires better data. Current polymer-brush data sets commonly contain polymer identity, dry thickness, and application performance but much less information about the operating interface. Future data sets should connect molecular identity and synthesis with state-resolved interfacial descriptors. Operating conditions and measurement protocols must be retained for these variables. Raw observables and inferred state variables should also remain distinguishable, together with the model or assumption used to connect them. Such records would preserve the pathway from design to state to function, rather than reducing each experiment to an isolated property value.

Better data alone do not solve the problem. Models should use polymer physics, molecular simulation, and interfacial thermodynamics to constrain which relationships are physically plausible. Scaling theory and self-consistent field models can define accessible conformational regimes. Electrostatic and charge regulation models can constrain ion partitioning. Molecular simulations can provide local hydration, coordination, and transport information. Machine learning can then identify relationships that are difficult to prescribe analytically. The aim is not to force every prediction into an existing theory but to use physical knowledge to define the prediction domain, expose inconsistencies, and distinguish transferable relationships from data set-specific correlations.

The experimental design must also change. Most current studies optimize or predict a single end point after the polymer brush has already been synthesized. A more informative strategy would select experiments that improve performance while also testing the reason for the improvement. For example, two conditions may produce similar protein resistance through different combinations of chain crowding and hydration. An informative next experiment would distinguish between these states rather than simply searching for a still lower adsorption value. The same principle applies to lubrication, separation, sensing, and particle assemblies. Practical optimization will also be multiobjective, balancing function with stability, permeability, regenerability, mechanical resistance, and synthetic accessibility.

Active learning provides a route to making these decisions sequentially. A model can identify regions of high uncertainty, competing physical explanations, or promising performances. Automated synthesis can execute the selected conditions, while state-resolved characterization determines whether the intended brush structure and interfacial state were actually produced. Functional testing then establishes how that state affects the performance. The combined results update the next experimental decision. Importantly, the loop should be closed through the measured interfacial state rather than through the performance alone.

A mature framework for interfacial intelligence should integrate provenance-aware literature data, molecular representation, polymer physics, molecular simulation, controlled synthesis, state-resolved characterization, and adaptive experimentation (Figure 7). Molecular design and processing define the range of brush structures that can be realized, while the operating conditions determine the corresponding interfacial states. State-resolved measurements and molecular simulations then establish how these states are related to the functional performance. Polymer physics should further constrain the physically accessible state space and generate testable predictions of how the interfacial state responds to controlled perturbations. New experiments can test these predictions, distinguish competing physical interpretations, and update the underlying models. Automation can accelerate this iterative process, but its role is to support rather than replace the physical understanding. The ultimate goal is therefore not simply faster performance optimization but a closed-loop system that continuously links design, realized interfacial state, function, physical interpretation, and experimental refinement.

**Figure 7:**
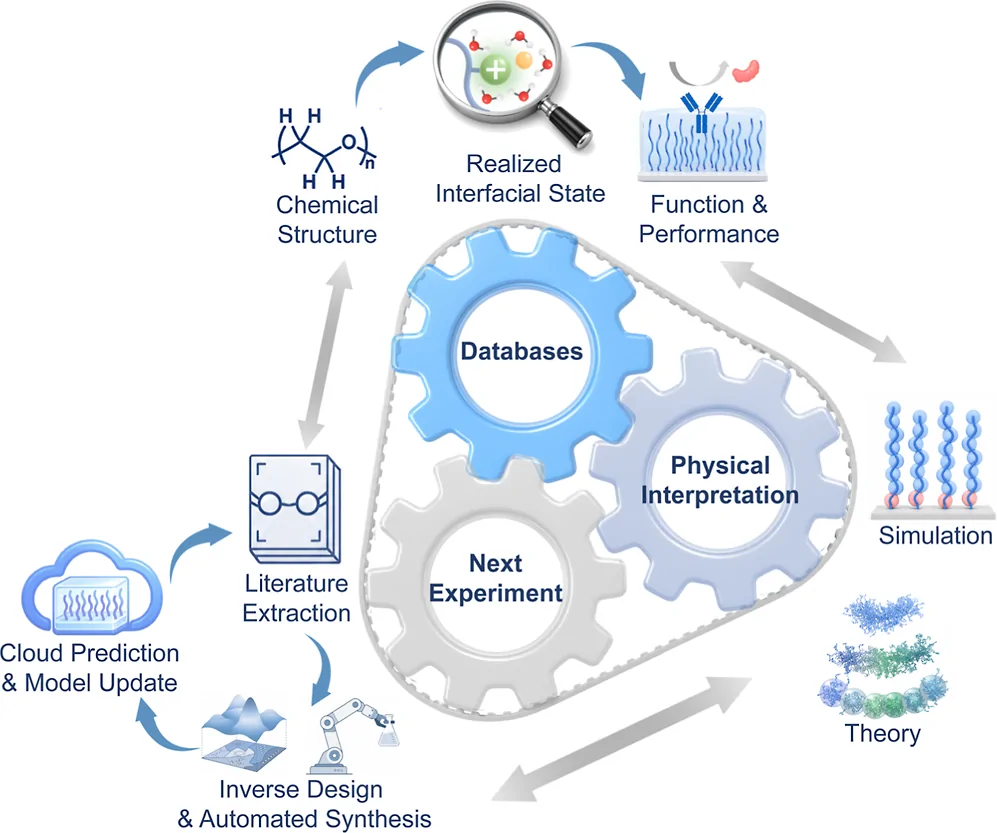
Conceptual roadmap toward closed-loop interfacial intelligence for polymer brushes. A closed-loop framework integrates literature extraction, polymer-brush databases, machine learning, polymer physics, molecular simulation, state-resolved characterization, autonomous investigation, inverse design, and automated synthesis. Molecular design and processing determine the brush structure, while operating conditions establish the realized interfacial state, which in turn governs functional performance. Importantly, the loop is closed through the measured interfacial state rather than through performance alone. Physics-grounded models relate state to function and generate testable predictions of how the interface should respond to controlled perturbations. Experimental outcomes then update the underlying representation and model and guide the next experiment, enabling a shift from end point optimization toward experimentally accountable and transferable interfacial design.
